# A Learnable Entropy-Power Scaling Transform with a Radial Basis Function Network for Electroencephalography-Based Familiar and Unfamiliar Face Classification

**DOI:** 10.3390/e28080866

**Published:** 2026-08-01

**Authors:** Chengyang Yan, Yazhou Zhao, Weidong Zhou, Guoyang Liu

**Affiliations:** 1 School of Integrated Circuits, Shandong University, Jinan 250101, China; 2 Shenzhen Research Institute of Shandong University, Shenzhen 518000, China; 3 Tandon School of Engineering, New York University, New York, NY 10012, USA; 4 Key Laboratory of Social Computing and Cognitive Intelligence, Dalian University of Technology, Ministry of Education, Dalian 116024, China

**Keywords:** EEG, familiar and unfamiliar face recognition, entropy power, learnable scaling transform, radial basis function network

## Abstract

Familiar and unfamiliar face recognition is an important cognitive process with potential applications in brain-computer interface (BCI) systems and neurocognitive assessment. Classification of familiar and unfamiliar faces based on electroencephalography (EEG) remains challenging because discriminative information is distributed across multiple frequency bands, temporal windows, and scalp channels. This study proposes LEPST-RBFNet, which combines a Learnable Entropy-Power Scaling Transform (LEPST) and a radial basis function (RBF) network for EEG-based familiar and unfamiliar face classification. The model first segments the EEG into multi-scale time-frequency segments and then extracts local standard-deviation features. After that, a learnable entropy-power-inspired scaling transformation is applied using the LEPST module to obtain adaptive local time-frequency EEG entropy features. The transformed features are classified by an RBF prototype module with learnable centers and an adaptive kernel-width parameter. Experiments using a five-fold leave-one-block-out validation protocol show that LEPST-RBFNet achieves a superior average classification accuracy of 73.60%. Ablation and visualization analyses further indicate that the proposed model provides a competitive and interpretable framework for EEG-based familiar and unfamiliar face recognition.

## 1. Introduction

Familiar and unfamiliar face recognition is a fundamental cognitive function in daily social interaction. Recognizing a familiar person from facial appearance is not merely a low-level visual task but also involves the retrieval of identity-specific semantic knowledge, autobiographical associations, and recognition-related decision processes. Classical cognitive models of face recognition have distinguished structural face encoding from later stages of face identity recognition and person knowledge retrieval [1]. Neurocognitive studies further suggest that face perception is supported by a distributed neural system, in which core visual face-processing regions interact with extended systems related to memory, emotion, and social cognition [2,3,4]. Compared with unfamiliar faces, familiar faces are usually processed more robustly and efficiently because repeated real-life exposure may establish stable identity representations across changes in viewpoint, illumination, expression, and context [5,6,7]. Therefore, objective discrimination between familiar- and unfamiliar-face-related brain responses is valuable for understanding human face cognition and for developing cognitive-state decoding and brain-computer interface (BCI) systems.

Electroencephalography (EEG) provides a non-invasive, low-cost, and temporally precise way to record neural responses during face perception and recognition, making it suitable for cognitive-state monitoring and BCI applications. Event-related potential (ERP) studies have shown that faces evoke characteristic components such as the N170, which is closely related to early structural face processing [8,9]. However, familiarity-related EEG responses are not limited to early visual processing. Familiar face recognition can involve later cognitive processes such as memory retrieval, identity association, and recognition-related decision formation. These processes may be reflected not only in transient evoked components but also in sustained spatio-temporal EEG patterns during the stimulus and recall period [10,11]. Recent EEG and ERP studies further indicate that the neural dynamics of familiar face recognition may involve multiple processing stages and can be decoded using multivariate or classifier-based analyses [7,10,12]. These findings suggest that EEG-based familiar and unfamiliar face decoding may provide useful information for BCI-oriented cognitive assessment and human-computer interaction.

Several EEG-based familiar and unfamiliar face classification methods have been proposed. Early work showed that familiar and unfamiliar face responses can be classified from EEG signals by selecting discriminative time intervals and channel information [13]. Liu et al. further introduced differential entropy features for EEG-based familiar and unfamiliar face classification and demonstrated the feasibility of classifying familiar and unfamiliar faces using time-frequency EEG representations [14]. More recently, a study proposed a filter-bank differential entropy (FBDE) framework combined with a Gaussian-kernel support vector machine (SVM) for EEG-based familiar and unfamiliar face classification [15]. The results demonstrated that EEG signals contain discriminative information for familiar and unfamiliar face recognition and that such information is distributed across temporal, frequency, and spatial domains. In addition to handcrafted feature-based methods, recent studies have also explored deep learning models and larger EEG datasets for familiar and unfamiliar face recognition [16,17].

Although these studies have demonstrated the feasibility of EEG-based familiar and unfamiliar face recognition, previous methods still have several limitations. First, although feature engineering models have fewer parameters and better generalization, handcrafted EEG features rely on fixed transformations, leading to suboptimal performance. For example, entropy-based or logarithmic transformations are usually predefined and applied uniformly to all frequency bands and temporal windows. Differential entropy has been shown to be effective in EEG feature representation [14,15,18], but fixed transformations may not be optimal because EEG features from different time-frequency components can have different statistical scales, noise characteristics, and discriminative strengths. Second, conventional classifiers often separate feature extraction from classification. Although SVM-based methods are effective for small EEG datasets [19,20], the feature representation itself is not adaptively transformed according to the final classification objective. Third, general deep learning models, such as convolutional neural networks (CNNs), may learn discriminative representations automatically [21,22,23]. However, they usually contain a larger number of trainable parameters and often require large-scale datasets to achieve stable generalization. In small EEG datasets, over-parameterized deep models are more likely to overfit and may learn subject-specific noise rather than task-related neural patterns [20,23]. Therefore, in EEG-based familiar and unfamiliar face classification and related BCI applications, compact and interpretable models are especially important.

These limitations reveal a research gap in EEG-based familiar and unfamiliar face classification: existing methods still lack a compact framework that can simultaneously support adaptive time-frequency feature transformation, competitive classification performance, and interpretable model-derived feature weights. Conventional feature-engineering methods are usually interpretable but rely on fixed hand-crafted transformations, whereas general deep learning models can learn discriminative representations but often require larger datasets and provide limited interpretability of time-frequency contributions. Therefore, it remains challenging to achieve adaptivity, compactness, and interpretability within a unified framework for few-shot EEG feature learning.

To address this gap, this study proposes LEPST-RBFNet, which combines a Learnable Entropy-Power Scaling Transform (LEPST) and a radial basis function (RBF) network for EEG-based familiar and unfamiliar face classification. The proposed method first extracts local standard-deviation features from multi-channel EEG signals after time-frequency window selection. Previous EEG-based recognition studies have shown that differential entropy features can effectively characterize task-related EEG states. In particular, differential entropy features have achieved strong performance in EEG-based emotion recognition, and differential-entropy-based features have also been used in EEG-based familiar and unfamiliar face classification [14,15,24]. These findings motivate the use of entropy-related representations for the present face classification task. Accordingly, the proposed LEPST module introduces an entropy-power-inspired learnable scale-power transformation to adaptively adjust local time-frequency EEG features. The entropy-power formulation further links differential entropy with the variance-related amplitude scale of EEG segments under the Gaussian approximation. This relationship motivates LEPST to transform local standard-deviation features using learnable scale-power parameters, rather than applying a fixed entropy or logarithmic feature transformation. The resulting LEPST module learns time-frequency-specific scaling parameters according to the classification objective, thereby allowing discriminative EEG components to be selectively preserved or compressed. The transformed features are further normalized and fed into an RBF prototype classifier, which maps high-dimensional EEG features into a nonlinear similarity space using learnable prototype centers.

The main contributions of this study can be summarized as follows:

- We propose a Learnable Entropy-Power Scaling Transform for EEG-based familiar and unfamiliar face classification. Unlike fixed differential-entropy or logarithmic features, the proposed LEPST module introduces learnable time-frequency-specific scale-power parameters, thereby enabling adaptive EEG feature transformation.
- We develop a compact and interpretable LEPST-RBFNet framework by combining multi-scale time-frequency feature extraction, LEPST transformation, and an RBF prototype classifier. Compared with general deep learning models, the proposed framework provides model-derived time-frequency scaling parameters and prototype-based similarity representation, which improves interpretability.
- We evaluate the proposed LEPST-RBFNet on our collected EEG dataset using a five-fold leave-one-block-out (LOBO) validation protocol. The experimental results, ablation studies, statistical comparison, and visualization analyses show that the proposed model achieves competitive performance while providing interpretable time-frequency scaling parameters.

The remainder of this paper is organized as follows. Section 2 describes the EEG dataset used in this study. Section 3 introduces the overall architecture of LEPST-RBFNet and the proposed method, including time-frequency window selection, LEPST learning, and RBF classification. Section 4 describes the training and evaluation settings and reports the classification results and then discusses ablation experiments, visualization and interpretation analysis, and comparison with existing studies. Section 5 summarizes the main findings, discusses limitations, and outlines future work.

## 2. Materials

We collected an EEG dataset for familiar and unfamiliar face recognition. The dataset contained multi-channel EEG recordings from ten healthy volunteers aged between 18 and 25 years. All participants had normal or corrected-to-normal vision. The dataset included seven male and three female participants, and each participant completed five experimental blocks under the familiar- and unfamiliar-face-recall paradigm used in the present work. The EEG data collection was approved by the Ethics Committee of Qilu Hospital of Shandong University.

The face stimuli were selected from two image sources. The familiar-face candidate set consisted of 378 face photos of world-famous people collected from the public domain, whereas the unfamiliar-face set consisted of 1000 face photos generated by StyleGAN2 [25]. All images were in color, resized to 480×480 pixels, and manually checked to ensure that the face appeared near the center of the image. Before the experiment, each participant was asked to remove all face photos that were unfamiliar to them from the famous-face candidate set. After this personalized screening procedure, 100 familiar face photos and 100 unfamiliar face photos were selected to construct an individualized familiar and unfamiliar face database for each participant. The unfamiliar face images were selected without repetition and were not exposed to the participants before the experiment, which helped reduce prior familiarity effects and ensured that the unfamiliar class remained unknown to the participants.

Each trial consisted of a 2 s preparation stage, a 4 s face image presentation stage, and a 3 s resting stage. During the preparation stage, the word “Preparation” was displayed on the screen to remind participants to concentrate on the subsequent face recognition task. Participants were instructed to keep their hands on their knees and to avoid eye movements or blinking as much as possible during face presentation. During the 4 s image presentation stage, a randomly selected face image was shown on the screen. When the displayed face was familiar, participants were asked to recall information related to the person, such as the person’s name, identity, or known experiences, until the image disappeared. When the displayed face was unfamiliar, participants viewed the face without prior identity knowledge. After the image presentation stage, a blank white screen was displayed for 3 s as the resting period. For each participant, five experimental blocks were recorded, and each block contained 40 continuous trials with an equal number of familiar and unfamiliar face images randomly distributed across trials. Therefore, each participant contributed 200 trials under the paradigm used in this study. A short break of at least two minutes was provided between adjacent blocks, and the actual break duration was determined according to the mental state of the participant.

**Figure 1:**
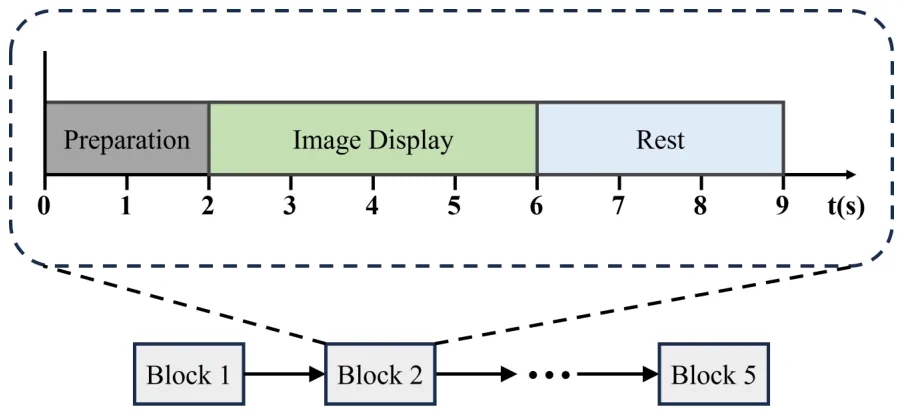
EEG data acquisition paradigm for the face recognition cognitive task. Each participant completed five experimental blocks, and each block contained 40 trials. In each trial, participants first underwent a 2 s preparation stage, followed by the face image stimulus presentation stage and a subsequent resting stage.

**Figure 2:**
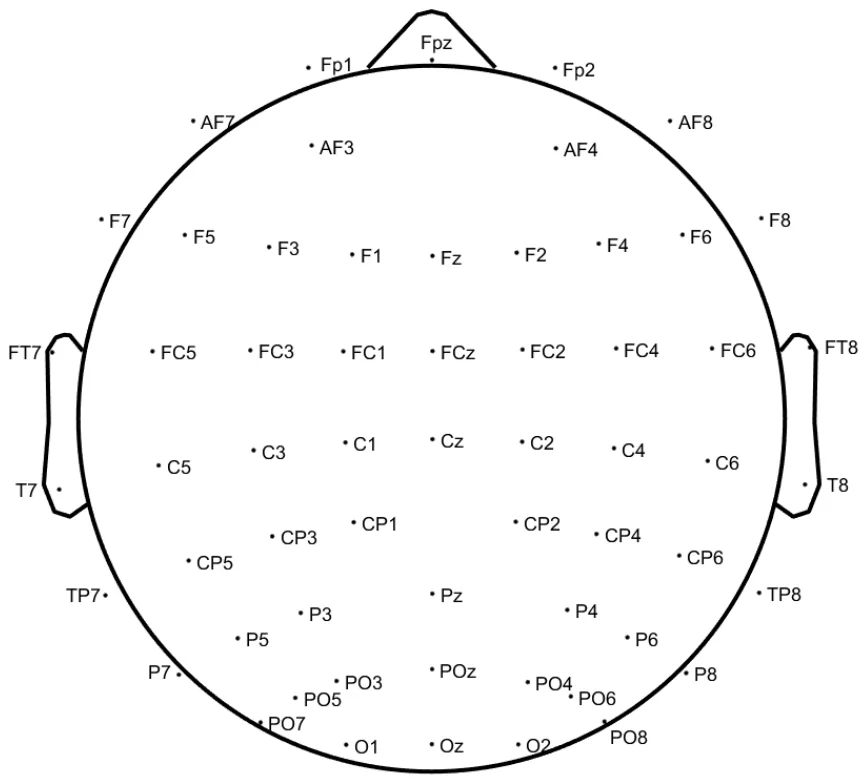
Spatial topological distribution of the 59 EEG electrodes used for signal acquisition.

EEG signals were recorded using a NeuSen-W64 EEG acquisition device at a sampling rate of 1000 Hz. A total of 59 scalp electrodes were placed according to the extended international 10–20 system. The reference electrode was located between Pz and Cz, and the ground electrode was located between Fpz and Fz. For preprocessing, the raw EEG signals were resampled from 1000 Hz to 250 Hz using the MATLAB R2021a resample function, which applies anti-aliasing filtering during resampling. For each trial, the 4 s EEG epoch corresponding to the face image presentation period was extracted for subsequent analysis, yielding 1000 sampling points for each EEG channel. Each extracted epoch was segmented into temporal windows, and each temporal window was filtered into 33 predefined frequency bands within 0.5–40 Hz using zero-phase band-pass filtering to suppress very-low-frequency drift and high-frequency noise while preserving task-related EEG rhythms. No additional manual trial rejection, channel rejection, or independent component analysis (ICA)-based eye-movement artifact correction was applied. All computational procedures were implemented using Python 3.10.20. Signal processing and feature extraction were mainly implemented using NumPy 2.2.5 and SciPy 1.15.3, and the proposed LEPST-RBFNet model was implemented using PyTorch 2.11.0. The experimental paradigm is shown in Figure 1, and the spatial distribution of the EEG electrodes is shown in Figure 2.

## 3. Methods

### 3.1. Overall Architecture

**Figure 3:**
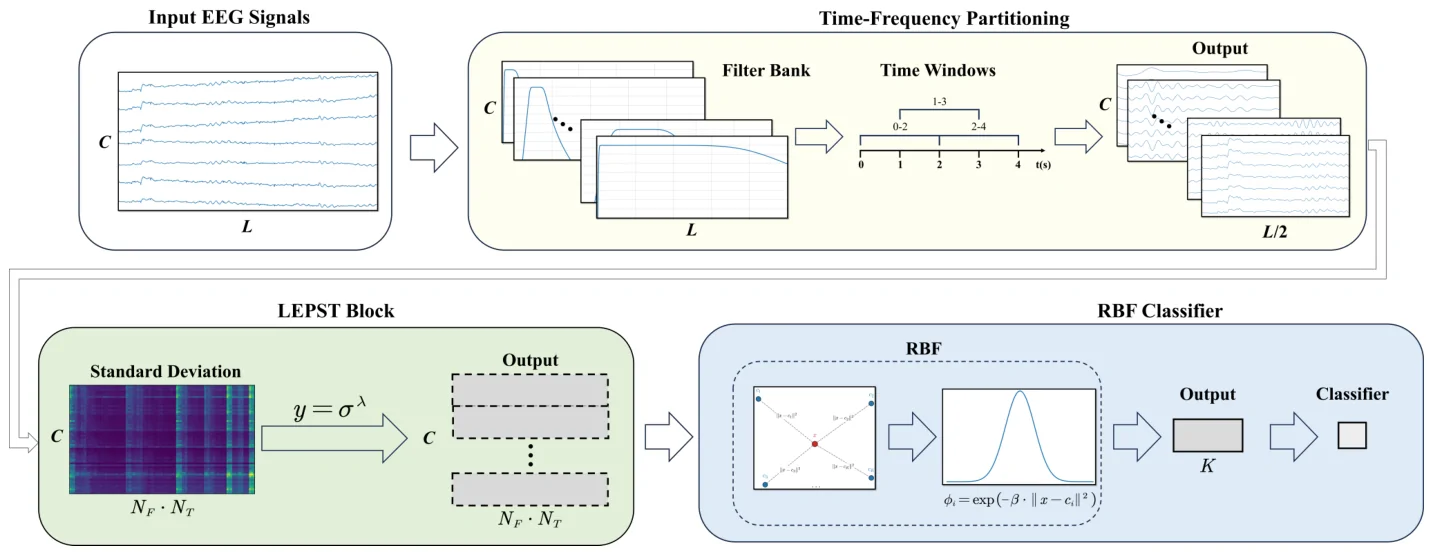
Overall architecture of the proposed LEPST-RBFNet model. Raw EEG signals are segmented in time and filtered across frequency bands to generate $N_{T}\times N_{F}$ local time-frequency segments. Local standard-deviation features are extracted from each channel and time-frequency segment, transformed by the LEPST learning block, and then fed into the RBF classifier for familiar and unfamiliar face classification.

The overall architecture of the proposed LEPST-RBFNet is shown in Figure 3, and the detailed network architecture is summarized in Table 1. The model receives multi-channel EEG trials as input and first performs time-frequency window selection. Specifically, the EEG signal is divided into $N_{T}$ temporal windows and filtered into $N_{F}$ frequency bands, forming $N_{T}\times N_{F}$ local time-frequency selections. For each selected segment, local standard-deviation features are extracted across *C* EEG channels, where C=59 in this study.

The extracted standard-deviation features are then fed into the LEPST learning block. In this block, each time-frequency feature is transformed by a learnable scale-power function $y=\sigma^{\lambda }$, where σ denotes the local standard-deviation feature and λ is the learnable scale-power parameter derived from the entropy-power formulation under the Gaussian assumption. After LEPST transformation, the features are organized as *C* channel-wise $N_{F}\times N_{T}$ feature representations. These representations are then flattened and fed into the RBF classifier, which maps the transformed EEG features into a prototype similarity space and outputs the final binary prediction.

The inference procedure of LEPST-RBFNet is summarized in Algorithm 1.
**Algorithm 1:** Inference procedure of LEPST-RBFNet
**Input:** A 4 s EEG epoch $X\in R^{C\times L}$ with C=59 and L=1000, together with the learned model parameters $\lambda_{f,t}$, ${\left\{c_{i}\right\}}_{i=1}^{K}$, β, w, and *b*.
**Output:** Predicted class label $\hat{y}\in \left\{\mathrm{familiar},\mathrm{unfamiliar}\right\}$.

1: Segment X into three 2 s temporal windows with 50% overlap.

2: for each temporal window $t=1,\ldots ,N_{T}$ do

3:    Apply the 33-band multi-scale zero-phase filter bank within 0.5–40 Hz.

4:    for each frequency band $f=1,\ldots ,N_{F}$ do

5:       for each EEG channel c=1,…,C do

6:          Compute local variance $v_{c,f,t}$.

7:          Compute local standard deviation $\sigma_{c,f,t}=\sqrt{v_{c,f,t}}$.

8:          Apply LEPST transformation $z_{c,f,t}=\sigma_{c,f,t}^{\lambda_{f,t}}$.

9:       end for

10:    end for

11: end for

12: Flatten $Z=\{z_{c,f,t}\}$ into a feature vector $x\in R^{5841}$.

13: Apply layer normalization to x.

14: for each RBF center i=1,…,K do

15:    Compute squared Euclidean distance $d_{i}\left(x\right)={∥x-c_{i}∥}_{2}^{2}$.

16:    Compute RBF response $\phi_{i}\left(x\right)=\exp\left(-\beta d_{i}\left(x\right)\right)$.

17: end for

18: Compute prediction logit $o=w^{T}\phi +b$.

19: Compute prediction probability p=sigmoid(o).

20: if p≥0.5 then

21:    Assign $\hat{y}$ to the positive class according to the predefined label encoding.

22: else

23:    Assign $\hat{y}$ to the negative class according to the predefined label encoding.

24: end if


### 3.2. Time-Frequency Window Selection and Feature Extraction

Each 4 s EEG epoch corresponding to the face image presentation period was segmented into three 2 s temporal windows with 50% overlap, namely 0–2 s, 1–3 s, and 2–4 s. This overlapping temporal-window design allows the model to capture local EEG responses from different stages of the stimulus period while maintaining sufficient samples within each window for stable feature estimation. The time-window segmentation, filter-bank processing, and local standard-deviation feature extraction were implemented using NumPy 2.2.5 and SciPy 1.15.3.

For each temporal window, a multi-scale filter-bank scheme was applied over the frequency range of 0.5–40 Hz. The filter bank included one full-band window, overlapping 4 Hz windows with a 2 Hz step, overlapping 8 Hz windows with a 4 Hz step, and overlapping 16 Hz windows with an 8 Hz step, resulting in 33 frequency bands in total. This multi-scale design enables the feature extraction procedure to represent both broad spectral energy distributions and relatively local frequency-specific variations. In addition, by summarizing each local time-frequency segment with a statistical feature rather than directly using the raw time samples, the original sample-level EEG sequence can be converted into a compact structured feature representation. This reduces the dimensionality of the input representation and helps limit the number of trainable parameters in the subsequent learning modules. Zero-phase filtering was applied to preserve temporal information and avoid phase distortion during band-pass filtering.

Let *C*, *L*, $N_{F}$, and $N_{T}$ denote the number of EEG channels, the number of sampling points in one EEG epoch, the number of frequency bands, and the number of temporal windows, respectively. In this study, C=59, L=1000, $N_{F}=33$, and $N_{T}=3$. Let $x_{c,f,t}\left(n\right)$ denote the filtered EEG signal from channel *c*, frequency band *f*, and temporal window *t*. For each channel and each time-frequency selection, the local variance was first calculated as

(1) $$v_{c,f,t}=\frac{1}{L_{t}-1}\sum_{n=1}^{L_{t}}{\left(x_{c,f,t}\left(n\right)-{\bar{x}}_{c,f,t}\right)}^{2},$$

where $L_{t}$ is the number of sampling points in temporal window *t* and ${\bar{x}}_{c,f,t}$ is the mean value within the corresponding segment. Then, a local standard-deviation feature was obtained by

(2) $$\sigma_{c,f,t}=\sqrt{v_{c,f,t}}.$$

As a result, each trial was represented by a structured local standard-deviation feature tensor with size $C\times N_{F}\times N_{T}$, corresponding to EEG channels, frequency bands, and temporal windows. In this study, the original preprocessed EEG epoch contained 59×1000 sample-level values, whereas the extracted feature tensor had a size of 59×33×3. This transformation converts the raw sample-level EEG sequence into a compact time-frequency statistical representation, which preserves local temporal and spectral information while reducing the dimensionality of the data passed to the subsequent LEPST learning block.

### 3.3. Learnable Entropy-Power Scaling Transform

Previous EEG-based recognition studies, including emotion recognition and familiar/unfamiliar face classification, have shown that differential entropy features can effectively characterize task-related EEG states [14,15,24]. Motivated by this observation, the proposed LEPST module reformulates local EEG features from the perspective of entropy power. Compared with directly using differential entropy as a fixed logarithmic feature, entropy power provides a theoretical bridge between information-theoretic entropy and the local amplitude scale of EEG segments. Different from conventional entropy-based EEG feature extraction methods that compute fixed entropy values before classification, LEPST converts the entropy-power relationship into a learnable scale-power transformation, allowing the time-frequency-specific parameters to be optimized jointly with the classifier.

For a continuous random variable *X*, the entropy power is defined as

(3) $$N\left(X\right)=\frac{1}{2\pi e}\exp\left(2h(X)\right),$$

where h(X) denotes the differential entropy of *X* [26].

Let f(x) denote the probability density function obtained from the amplitude distribution of a single-channel EEG segment. The differential entropy of *X* is defined as

(4) h(X)=−∫f(x)logf(x)dx.

In this study, the filtered EEG segment within each local time-frequency window is modeled as an approximately Gaussian random variable. Early EEG distribution studies have reported that scalp EEG amplitude distributions can exhibit Gaussian-like behavior under certain recording conditions [27], which provides empirical support for using a Gaussian-based approximation in the present entropy-power formulation. To further examine whether the segmented and filtered EEG recordings in the familiar and unfamiliar face classification task approximately satisfy this assumption, we conducted the Kolmogorov-Smirnov normality test on more than two million single-channel EEG segments at the 5% significance level. For each segment, the normality assumption was tested according to its amplitude distribution. The results showed that more than 80% of the analyzed EEG segments did not reject the normality assumption. Therefore, the Gaussian-based differential entropy formulation is considered a reasonable approximation for extracting EEG features in the present task.

When $X∼N(\mu ,\sigma^{2})$, its differential entropy can be written as

(5) $$h\left(X\right)=\frac{1}{2}\log\left(2\pi e\sigma^{2}\right).$$

Substituting this expression into the definition of entropy power gives

(6) $$N\left(X\right)=\sigma^{2}.$$

Therefore, under the Gaussian approximation, entropy power is equivalent to the signal variance. Based on this property, the entropy-power representation can be further generalized by introducing a learnable scale-power parameter:

(7) $$N{\left(X\right)}^{k}={\left(\sigma^{2}\right)}^{k}=\sigma^{2k}=\sigma^{\lambda },$$

where λ=2k denotes the equivalent learnable scale-power parameter. This formulation converts the entropy-power representation into a learnable power transformation of the local standard-deviation feature. As a result, the model can adaptively adjust the scaling behavior of different time-frequency EEG components instead of relying on a fixed entropy-based or logarithmic transformation.

In this study, the local standard-deviation feature $\sigma_{c,f,t}$ is obtained from the variance feature $v_{c,f,t}$, where *c*, *f*, and *t* denote the EEG channel, frequency band, and temporal window, respectively. The proposed LEPST transformation is formulated as

(8) $$z_{c,f,t}=\sigma_{c,f,t}^{\lambda_{f,t}},$$

where $\lambda_{f,t}$ is a learnable scale-power parameter. The parameter $\lambda_{f,t}$ is shared across channels but learned independently for each frequency band and temporal window. This design reduces the number of trainable parameters and encourages the learned scale-power parameters to reflect time-frequency-specific discriminative characteristics rather than electrode-specific fluctuations. Therefore, LEPST can adaptively adjust the nonlinear scaling of EEG features from different time-frequency components while maintaining a compact parameterization.

A smaller $\lambda_{f,t}$ compresses the dynamic range of the corresponding time-frequency feature, thereby reducing its discriminative sensitivity in the transformed feature space. In contrast, a larger $\lambda_{f,t}$ preserves or amplifies feature differences, suggesting that the corresponding time-frequency component contributes more strongly to classification. It should be noted that the learned $\lambda_{f,t}$ values are not direct physiological power measurements. Instead, they are model-derived discriminative scaling parameters that indicate how strongly each time-frequency component is preserved or compressed during feature transformation.

The LEPST scale-power parameters are optimized by backpropagation. In this study, $\sigma_{c,f,t}$ denotes the local standard-deviation feature. To ensure that the logarithmic term is well-defined in the implemented LEPST transformation, $\sigma_{c,f,t}$ is constrained to be positive by adding a small positive constant when necessary. Therefore, the LEPST transformation can be rewritten as

(9) $$z_{c,f,t}=\sigma_{c,f,t}^{\lambda_{f,t}}=\exp\left(\lambda_{f,t}\log\sigma_{c,f,t}\right).$$

Therefore, the derivative of the transformed feature with respect to the corresponding scale-power parameter is

(10) $$\frac{\partial z_{c,f,t}}{\partial \lambda_{f,t}}=\exp\left(\lambda_{f,t}\log\sigma_{c,f,t}\right)\log\sigma_{c,f,t}=z_{c,f,t}\log\sigma_{c,f,t}.$$

Here, $L_{\mathrm{BCE}}$ denotes the binary cross-entropy (BCE) loss with logits used for the binary familiar/unfamiliar classification task. Because $\lambda_{f,t}$ is shared across EEG channels for the same frequency band and temporal window, the classification-loss gradient with respect to $\lambda_{f,t}$ is accumulated over all channels according to the chain rule:

(11) $$\frac{\partial L_{\mathrm{BCE}}}{\partial \lambda_{f,t}}=\sum_{c=1}^{C}\frac{\partial L_{\mathrm{BCE}}}{\partial z_{c,f,t}}\frac{\partial z_{c,f,t}}{\partial \lambda_{f,t}}=\sum_{c=1}^{C}\frac{\partial L_{\mathrm{BCE}}}{\partial z_{c,f,t}}z_{c,f,t}\log\sigma_{c,f,t}.$$

When the regularization term on the LEPST scale-power parameters is included, the total training loss is

(12) $$L=L_{\mathrm{BCE}}+\alpha \frac{1}{N_{F}N_{T}}\sum_{f=1}^{N_{F}}\sum_{t=1}^{N_{T}}{\left(\lambda_{f,t}-\lambda_{0}\right)}^{2}.$$

Thus, the gradient of the total loss with respect to $\lambda_{f,t}$ is

(13) $$\frac{\partial L}{\partial \lambda_{f,t}}=\sum_{c=1}^{C}\frac{\partial L_{\mathrm{BCE}}}{\partial z_{c,f,t}}z_{c,f,t}\log\sigma_{c,f,t}+\frac{2\alpha }{N_{F}N_{T}}\left(\lambda_{f,t}-\lambda_{0}\right).$$

This gradient shows that each LEPST parameter is updated according to both the discriminative feedback from all EEG channels within the same time-frequency component and the regularization constraint around the reference value $\lambda_{0}$. Therefore, the LEPST scale-power parameters can be optimized jointly with the subsequent normalization layer, RBF classifier, and linear output layer during network training.

### 3.4. RBF Classifier

**Figure 4:**
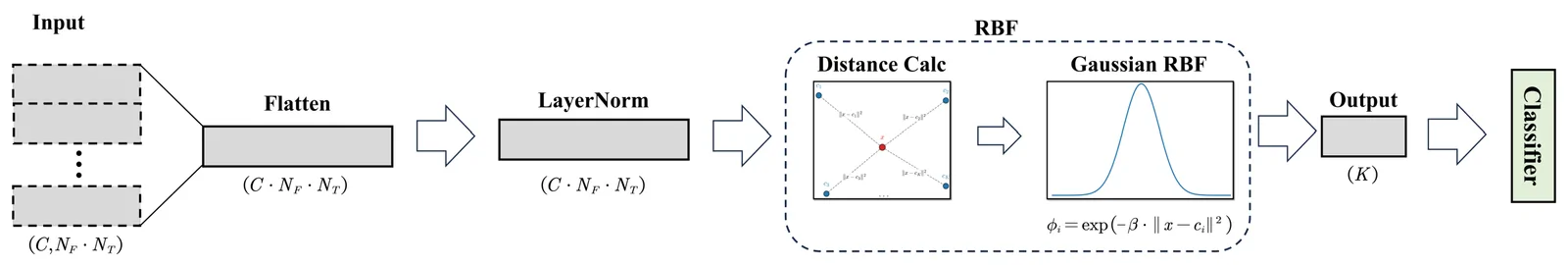
Internal structure of the RBF classifier. The flattened LEPST feature vector is first normalized using layer normalization. Squared Euclidean distances between the input feature and prototype centers are computed and converted into similarity responses by a Gaussian RBF kernel. The final prediction is generated by a linear output head.

After LEPST transformation, the feature tensor is flattened into a feature vector and then normalized using layer normalization. The normalized feature vector is fed into the RBF classifier, whose internal structure is shown in Figure 4. RBF networks use locally activated basis functions to map input features into a nonlinear similarity space. Compared with a purely linear classifier, this local-response mechanism allows the classifier to capture nonlinear class structures while retaining an interpretable prototype-based representation. Early studies on RBF networks related this architecture to multivariable interpolation and adaptive learning networks and showed that locally tuned processing units can be used for both classification and function approximation problems [28,29]. In addition, RBF networks have been theoretically studied from the perspectives of regularization and universal approximation, supporting their ability to model nonlinear input-output mappings [30].

Let $x\in R^{5841}$ denote the normalized feature vector and $c_{i}$ denote the *i*-th prototype center, where i=1,2,…,K. Each prototype center represents a reference point in the transformed EEG feature space. The squared Euclidean distance between the input feature and each prototype is calculated as

(14) $$d_{i}\left(x\right)={∥x-c_{i}∥}_{2}^{2}.$$

The RBF response is then obtained using a Gaussian kernel:

(15) $$\phi_{i}\left(x\right)=\exp\left(-\beta d_{i}\left(x\right)\right),$$

where β is a learnable kernel-width parameter. A larger RBF response indicates that the input EEG feature is closer to the corresponding prototype center in the normalized feature space. Therefore, the RBF layer converts the high-dimensional LEPST feature vector into a compact prototype-similarity representation. The final prediction logit is computed by a linear classification head:

(16) $$o=w^{T}\phi +b.$$

The binary prediction probability is obtained by applying the sigmoid function to *o*. In the proposed LEPST-RBFNet model, the number of RBF prototype centers is set to K=140. Combining the LEPST transformation, layer normalization, RBF similarity mapping, and linear output layer, the overall forward prediction of LEPST-RBFNet can be summarized as

(17) $$p=\mathrm{sigmoid}\left(w^{T}\phi \left(\mathrm{LN}\left(\mathrm{vec}(Z)\right)\right)+b\right),\;Z=\left\{z_{c,f,t}\right\}.$$

Here, vec(·) denotes vectorization, LN(·) denotes layer normalization, and ϕ(·) denotes the RBF similarity mapping.

The initialization of RBF prototype centers is important for stable training because the initial centers determine the early coverage of the EEG feature distribution. In the proposed model, farthest-point initialization was adopted. Feature vectors extracted from the training set were first obtained using the LEPST feature extractor and layer normalization. One training sample was randomly selected as the first center. The remaining centers were selected iteratively by choosing the sample with the largest minimum squared Euclidean distance to the already selected centers. This strategy encourages the initial centers to be distributed over different regions of the training feature space, reducing excessive center concentration and providing a more uniform initialization than random sampling. After initialization, the prototype centers are further updated during network training, allowing the RBF layer to adapt its similarity representation to the familiar and unfamiliar face classification objective.

## 4. Results and Discussion

### 4.1. Results

The model was trained using the Adam optimizer. The LEPST scale-power parameters were optimized with a learning rate of $1\times 10^{-4}$, while the layer normalization, RBF layer, and linear classifier were optimized with a learning rate of $1\times 10^{-3}$. A smaller learning rate was used for the LEPST scale-power parameters because $\lambda_{f,t}$ directly controls the nonlinear power transformation of the time-frequency EEG features. Rapid changes in $\lambda_{f,t}$ may substantially alter the feature distribution passed to the subsequent classifier and make it difficult for the RBF layer and linear output head to adapt stably. Therefore, a lower learning rate was adopted for the LEPST scale-power parameters to improve training stability, whereas a relatively larger learning rate was used for the classifier-related parameters to facilitate discriminative learning. The batch size was set to 160, and the model was trained for 800 epochs.

The BCE-with-logits loss was used as the classification loss. The $L_{2}$-type regularization term described in Section 3.3 was applied to prevent excessive deviation of the LEPST scale-power parameters. In the experiments, the regularization coefficient was set to $\alpha =10^{-3}$, and the reference value was set to $\lambda_{0}=0.5$. This regularization term was used to preserve the adaptive ability of the LEPST transformation while improving training stability.

A five-fold LOBO cross-validation strategy was adopted. In each fold, one block was used as the testing set and the remaining four blocks were used for training. The reported performance was averaged across folds and subjects.

In Table 2, Block 1–Block 5 correspond to the five testing folds under the LOBO protocol, where each block was used once as the testing set. The average accuracies across subjects for Blocks 1–5 were 71.50%, 71.75%, 75.50%, 75.00%, and 74.25%, respectively, indicating that the classification performance varied across testing blocks. The Max column reports the highest block-level accuracy obtained by each subject across the five testing folds. The increase in average accuracy from Block 1 to Block 3 may be related to improved task familiarity or more stable engagement after participants adapted to the experimental paradigm, whereas the slight decrease from Block 3 to Block 5 may be associated with fatigue, reduced attention, or temporal nonstationarity of EEG signals. Therefore, LEPST-RBFNet showed effective decoding capability across participants, but performance across blocks may still be affected by participant state and EEG nonstationarity.

At the subject level, the proposed model achieved an overall mean accuracy of 73.60% across ten subjects. Among all subjects, Subject 2 obtained the highest mean accuracy of 92.00%, indicating that the EEG responses of this participant contained highly discriminative patterns for familiar and unfamiliar face classification. Subject 3 obtained the lowest mean accuracy of 62.50%, suggesting that the classification performance varied across individuals. This inter-subject variability may be related to differences in face familiarity, cognitive recall strategy, and attention level. Overall, these results demonstrate that LEPST-RBFNet achieved competitive classification performance under the LOBO evaluation protocol.

**Figure 5:**
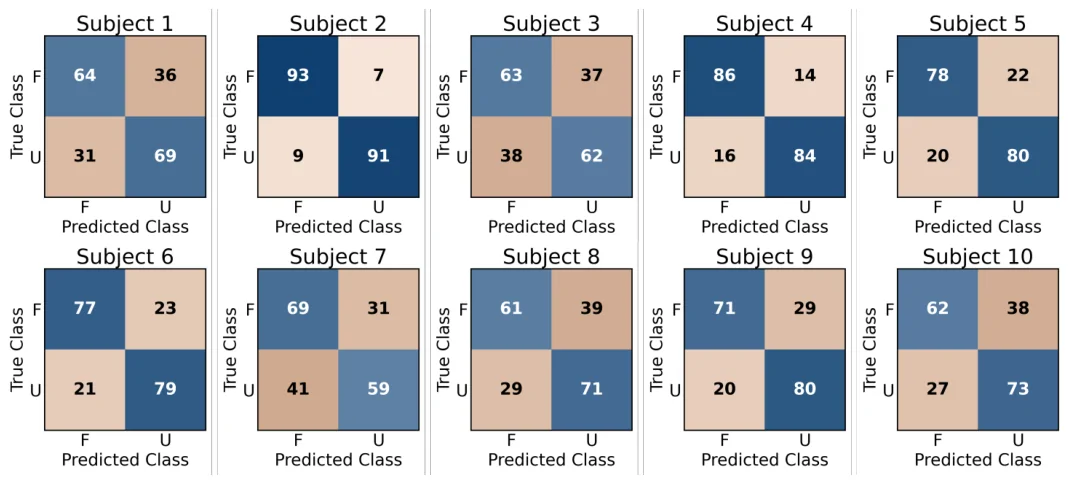
Confusion matrix of LEPST-RBFNet under the LOBO validation protocol for the familiar and unfamiliar face classification task.

To further illustrate the binary classification behavior of the proposed model, the confusion matrix of LEPST-RBFNet is shown in Figure 5. The diagonal elements represent correctly classified familiar and unfamiliar face trials, while the off-diagonal elements indicate misclassification between the two classes. The confusion matrix provides an intuitive overview of the error distribution and indicates that LEPST-RBFNet can distinguish EEG responses induced by familiar and unfamiliar face stimuli under the LOBO validation setting.

### 4.2. Ablation Experiments

#### 4.2.1. Ablation Study on Time-Frequency Window Selection

To investigate the influence of temporal window length and frequency window width on classification performance, a traversal experiment was first conducted. The temporal window lengths were set to 0.5 s, 1 s, 2 s, and 4 s, while the frequency window widths were set to 16 Hz, 8 Hz, 4 Hz, and 2 Hz. The experimental results showed that the model achieved the highest classification accuracy when the temporal window length was 2 s and the frequency window width was 8 Hz. Therefore, this parameter combination was adopted as the basis for subsequent experiments.

**Figure 6:**
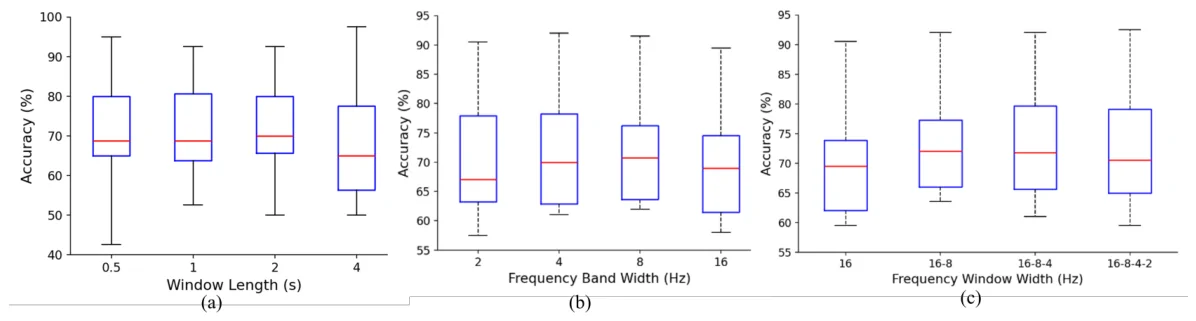
Effects of temporal window length, frequency window width, and multi-scale frequency-window combinations on the classification accuracy of LEPST-RBFNet. (**a**) Classification accuracy under different temporal window lengths with the frequency window width fixed at 8 Hz. (**b**) Classification accuracy under different frequency window widths with the temporal window length fixed at 2 s. (**c**) Classification accuracy under different multi-scale frequency-window combinations with the temporal window length fixed at 2 s.

Based on this setting, the effects of temporal window length, frequency window width, and multi-scale frequency-window combinations were further analyzed. First, under the condition that the frequency window width was fixed at 8 Hz, the classification accuracies obtained with different temporal window lengths were compared, as shown in Figure 6a. When the temporal window length was 0.5 s and 1 s, the accuracies were 71.85% and 71.90%, respectively, showing only a slight difference. The highest accuracy, 72.05%, was obtained when the temporal window length was 2 s. In contrast, when the temporal window length was increased to 4 s, the accuracy decreased to 68.25%, which was the lowest among the compared temporal-window settings. This result suggests that an excessively long temporal window is not beneficial for this task. Since a 4 s window covers the entire stimulus period, each trial provides only one temporal segment, reducing the number of local temporal observations that can be used by the model. Moreover, a long window may mix EEG responses from different cognitive stages, thereby weakening temporally specific discriminative information. By comparison, a 2 s window provides a better balance between temporal stability and temporal specificity.

Second, under the condition that the temporal window length was fixed at 2 s, four frequency window widths, namely 16 Hz, 8 Hz, 4 Hz, and 2 Hz, were compared, as shown in Figure 6b. The lowest accuracy, 69.95%, was obtained when the frequency window width was 16 Hz. This may be because an excessively wide frequency window provides only a small number of spectral selections and tends to merge neural activities from different frequency rhythms, thereby reducing frequency-specific discriminative information. Although a 2 Hz frequency window provides finer spectral resolution and generates more frequency selections, it did not yield the best performance. A very narrow frequency window may fragment useful spectral information into highly overlapping and redundant components. In addition, narrow-band filtering within a limited temporal window may produce less stable variance estimates, especially in low-frequency ranges where fewer oscillatory cycles are available. Therefore, an overly narrow frequency window may increase feature redundancy and noise sensitivity rather than improve classification performance. Overall, the 8 Hz frequency window achieved the best balance between spectral resolution and feature stability.

Furthermore, to evaluate the influence of multi-scale frequency-window combinations, several frequency-window combinations were compared under a fixed temporal window length of 2 s, as shown in Figure 6c. The results showed that combining multiple spectral scales improved the classification performance compared with using a single frequency-window width. Specifically, the combination of the full-band 40 Hz range with 16 Hz, 8 Hz, and 4 Hz frequency windows achieved the highest accuracy of 73.60%. This multi-scale design allows the model to simultaneously capture global spectral energy, medium-scale rhythmic patterns, and relatively fine-grained frequency-specific information. However, when 2 Hz frequency windows were further added, the classification accuracy began to decrease. This indicates that excessively fine spectral selection may introduce redundant and highly correlated features, increasing the risk of overfitting and reducing the robustness of the learned representation. Therefore, the final LEPST-RBFNet adopted the multi-scale frequency-window combination consisting of the full 40 Hz band, 16 Hz windows, 8 Hz windows, and 4 Hz windows.

#### 4.2.2. Ablation Study on the Classification Module

To verify the effectiveness of the classification module, the LEPST features were combined with different classification heads or activation functions, including a linear head, rectified linear unit (ReLU), and Gaussian error linear unit (GELU). As shown in Table 3, the RBF classification head achieved the highest average accuracy of 73.60 ± 9.90%. In contrast, the linear, ReLU, and GELU heads achieved average accuracies of 70.05 ± 11.17%, 71.75 ± 10.52%, and 71.25 ± 10.79%, respectively. These results indicate that the RBF prototype head can better model the nonlinear distribution of transformed EEG features.

**Figure 7:**
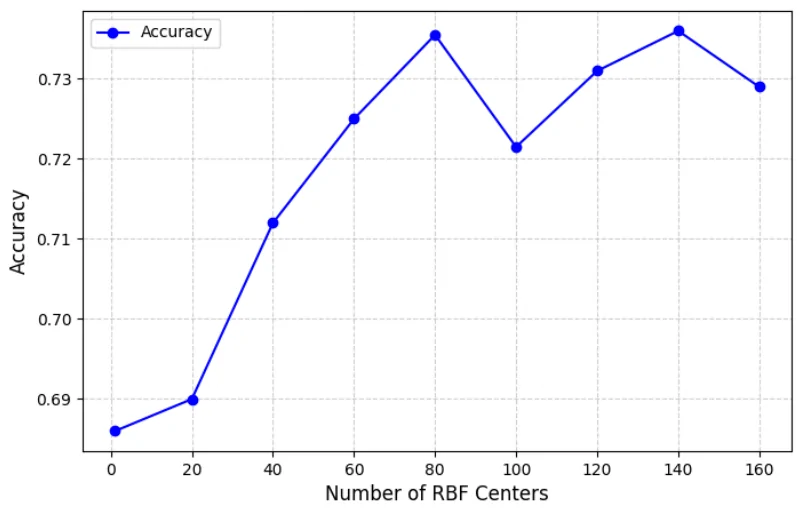
Effect of the number of RBF prototype centers on the classification accuracy of LEPST-RBFNet.

For the RBF-based classification module, the number of prototype centers directly determines the capacity of the nonlinear similarity representation. Therefore, different numbers of prototype centers were evaluated. As shown in Figure 7, the average accuracy increased from 68.60 ± 9.90% with one center to 73.60 ± 9.90% with 140 centers. When the number of centers was further increased to 160, the average accuracy decreased slightly to 72.90 ± 10.33%. These results suggest that too few prototypes are insufficient to represent the high-dimensional EEG feature distribution, whereas too many prototypes may introduce redundancy. Based on the experimental comparison, the number of RBF centers in the final LEPST-RBFNet model was set to K=140.

Different initialization strategies for RBF prototype centers were further compared. Random Samples initialized the prototype centers by randomly selecting training samples, representing a simple data-driven initialization strategy without clustering. K-Means is an unsupervised clustering method that partitions training features into a predefined number of clusters and uses the cluster centroids as prototype centers. Global K-Means initialized the RBF centers by applying K-Means clustering to all training features without considering class labels. Classwise K-Means performed K-Means separately within each class and then combined the resulting class-specific centers. Farthest-point initialization selected centers iteratively by choosing the sample with the largest minimum distance to the already selected centers, thereby encouraging the prototypes to cover the training feature distribution more broadly.

As shown in Table 4, farthest-point initialization achieved the highest average accuracy of 73.60 ± 9.90%. Random Samples, Global K-Means, and Classwise K-Means achieved average accuracies of 73.15 ± 10.32%, 73.05 ± 10.31%, and 73.15 ± 10.10%, respectively. The advantage of farthest-point initialization may be attributed to its ability to reduce excessive center concentration and provide a more uniformly distributed set of initial prototypes. Although Classwise K-Means uses label information by clustering samples within each class, its performance did not exceed that of farthest-point initialization. This may be because the number of training samples in each class is relatively limited under the LOBO setting, making class-specific clustering more sensitive to within-class variability and noise. In contrast, farthest-point initialization emphasizes global coverage of the training feature distribution, which may provide more robust initial prototypes for the subsequent adaptive RBF learning. However, the performance difference among initialization strategies was relatively small, suggesting that prototype initialization improves stability but is not the only factor determining classification performance.

#### 4.2.3. Ablation Study on Learnable Components

An ablation study was conducted to evaluate the contribution of the learnable components in LEPST-RBFNet. As shown in Table 5, fixing the LEPST scale-power parameters reduced the average accuracy from 73.60 ± 9.90% to 73.25 ± 9.80%. This result suggests that learning the scale-power parameters $\lambda_{f,t}$ provides a modest additional benefit by allowing the model to adaptively adjust the nonlinear transformation of different time-frequency EEG components. However, the performance difference between the fixed and learnable LEPST settings was relatively small. Although this improvement was small, the learnable LEPST module still achieved the best average performance among the tested variants.

In addition, fixing the RBF prototype centers after initialization reduced the average accuracy to 70.10 ± 9.73%, suggesting that adaptive prototype updating is important for constructing an effective similarity representation. Overall, these results show that both the learnable LEPST transformation and the trainable RBF prototype centers can effectively contribute to the final performance of LEPST-RBFNet.

Additionally, to further examine whether the entropy-related representation adopted in the proposed framework was suitable for the present task, we conducted an additional comparison with three alternative entropy measures, including Shannon entropy, amplitude entropy, and incremental entropy, under the same LOBO validation protocol. In this comparison, only the entropy-related feature formulation was replaced, while the remaining model structure and training settings were kept unchanged. As shown in Table 6, Shannon entropy, amplitude entropy, and incremental entropy achieved average accuracies of 60.10%, 55.50%, and 52.83%, respectively, whereas differential entropy achieved an average accuracy of 73.60%. These results indicate that the differential-entropy-based formulation was more suitable for the proposed LEPST-RBFNet framework than the other tested entropy measures.

### 4.3. t-SNE Feature Visualization

**Figure 8:**
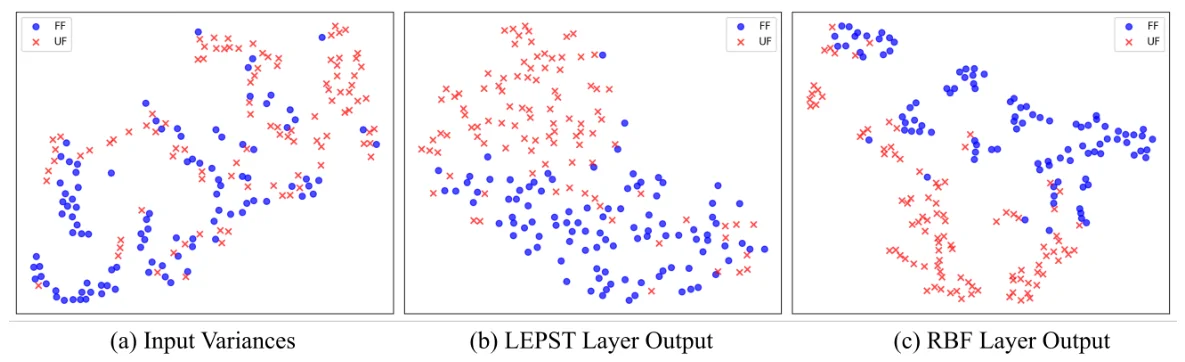
t-SNE visualization of feature distributions at different processing stages of LEPST-RBFNet. (**a**) Original standard-deviation features. (**b**) LEPST-transformed features. (**c**) RBF similarity representations. The progressive separation of the two classes indicates that the proposed model improves the discriminative structure of EEG features.

To further interpret the representation-learning behavior of LEPST-RBFNet, t-distributed Stochastic Neighbor Embedding (t-SNE) visualization was conducted as a post hoc analysis. t-SNE is a nonlinear dimensionality-reduction method that maps high-dimensional samples into a low-dimensional space while preserving local neighborhood relationships, making it suitable for visualizing the separability of learned feature representations [31]. In this study, t-SNE was used only to visualize the feature distributions at different processing stages and was not involved in model training or testing. As shown in Figure 8, the original standard-deviation features showed a relatively mixed distribution, indicating that familiar and unfamiliar face trials were difficult to separate directly in the original time-frequency feature space. This suggests that the raw local amplitude-scale features still contain overlapping class information and require further discriminative transformation.

After LEPST transformation, the two classes became more distinguishable. This observation suggests that the learnable scale-power parameters improved the discriminative scaling of time-frequency EEG features by preserving or amplifying informative components while compressing less useful variations. Therefore, the LEPST layer did not merely perform a fixed nonlinear transformation but adaptively reshaped the feature distribution according to the classification objective.

After RBF mapping, the class structure became more compact and better separated. This indicates that the RBF layer further transformed the high-dimensional LEPST features into a prototype-similarity representation, in which samples with similar discriminative patterns were mapped closer to relevant prototype centers. Compared with the original feature space, the RBF similarity space showed clearer class organization, suggesting that the RBF layer not only performs final classification but also contributes to representation refinement.

### 4.4. Frequency-Specific Interpretation

The learned LEPST scale-power parameters provide additional interpretability for the proposed model. For each subject, the LEPST layer learned one scale-power parameter $\lambda_{f,t}$ for each frequency band *f* and temporal window *t*. Since the LEPST transformation is defined as $z=\sigma^{\lambda }$, a larger λ value indicates that the feature differences of the corresponding time-frequency component are more strongly preserved or amplified after transformation. In contrast, a smaller λ value indicates stronger compression of feature differences. Therefore, the learned λ values can be used as model-derived discriminative scaling parameters to indicate how strongly different time-frequency components contribute to the transformed feature representation.

**Figure 9:**
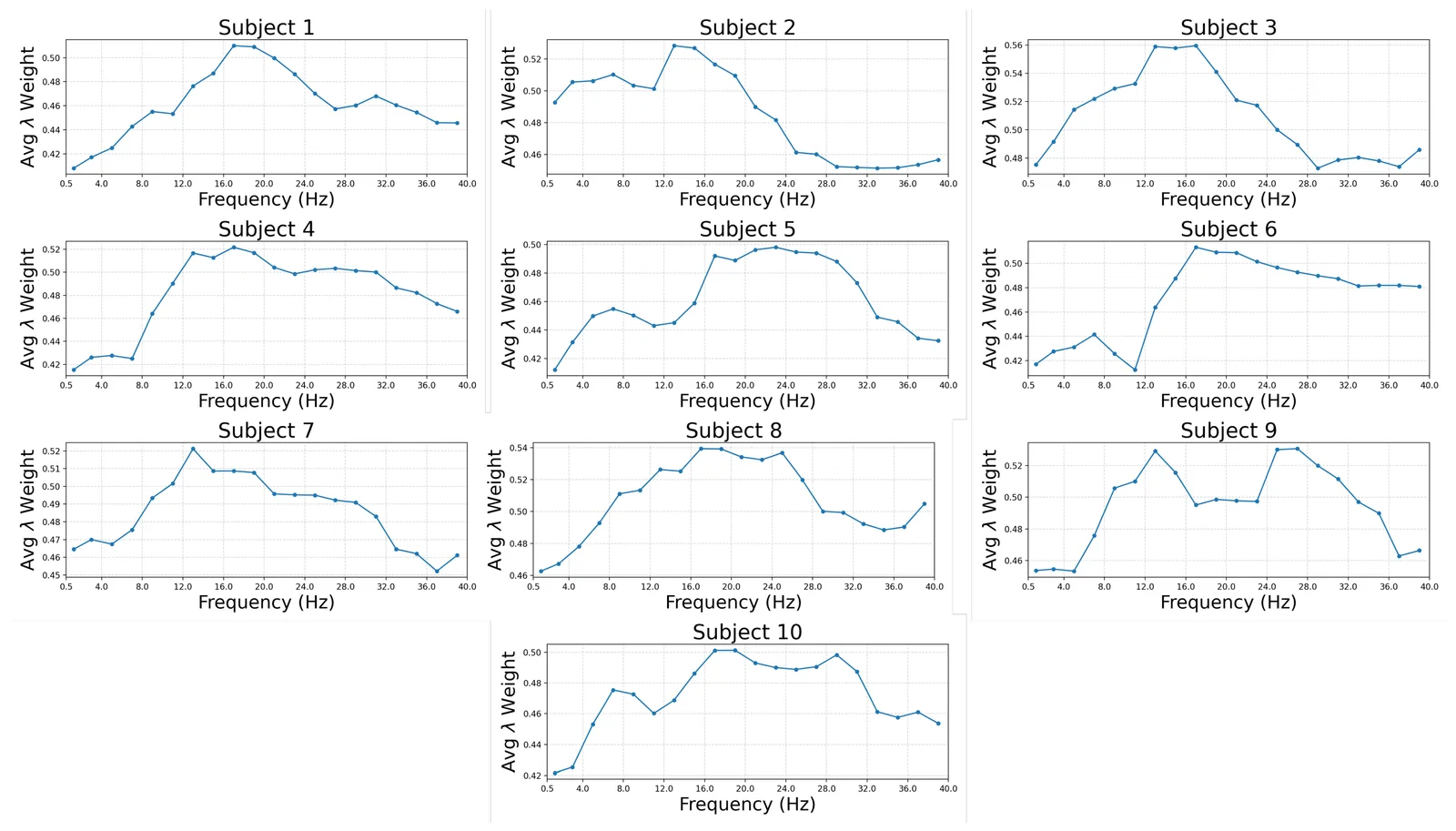
Frequency-wise distribution of the learned LEPST scale-power parameters λ for ten subjects. The parameters were first averaged across temporal windows for each filter-bank band and then mapped to the frequency axis. For overlapping frequency intervals, the displayed weight was calculated by averaging the weights of all filter bands covering that interval. Larger values indicate stronger preservation or amplification of feature differences, whereas smaller values indicate stronger compression. The learned weights show a middle-frequency-dominant pattern, with relatively higher values mainly concentrated in the beta range, commonly defined as 13–30 Hz.

To obtain the frequency-wise representation shown in Figure 9, the learned parameters were first averaged across the three temporal windows:

(18) $${\bar{\lambda }}_{f}=\frac{1}{N_{T}}\sum_{t=1}^{N_{T}}\lambda_{f,t},$$

where $N_{T}=3$. The resulting filter-bank-level weights were then projected onto the frequency axis. Because the filter-bank design contained overlapping frequency ranges, the weight of each frequency interval was calculated by averaging the weights of all filter bands covering that interval. Therefore, Figure 9 reflects the subject-wise frequency distribution of the learned LEPST weights. It should be noted that the learned λ values are not direct physiological power measurements. Instead, they are model-derived discriminative parameters that indicate how strongly each frequency component is preserved or compressed in the transformed feature space.

As shown in Figure 9, the frequency-wise distribution of the learned λ values showed a clear middle-frequency-dominant pattern: the weights were relatively low in the low-frequency and high-frequency ranges, whereas higher values were relatively concentrated in the beta-frequency range, commonly defined as 13–30 Hz. This result suggests that beta-band EEG components may contain more discriminative information for familiar and unfamiliar face classification in the proposed model.

This finding may be related to the cognitive demands of the familiar-face recognition task. Beta oscillations have been associated with attention, working memory, perceptual decision making, top-down control, and the maintenance of task-relevant neural states [32,33]. Recent studies on affective face processing and facial emotion recognition have also reported beta-band involvement in face-related social cognitive processing [34,35]. In the present task, participants needed to judge whether a presented face was familiar and, for familiar faces, access identity-related information. Therefore, the relatively higher LEPST weights in the beta range may indicate that beta-band EEG components carried task-relevant information for familiarity judgment in this experiment.

### 4.5. Comparison with Existing Studies

Table 7 summarizes the performance of LEPST-RBFNet and representative EEG-based classification methods evaluated on the same dataset under the same LOBO validation protocol. The evaluated methods include both conventional machine learning methods and recent deep learning models, such as EEGEncoder [36], Patched Brain Transformer (PBT) [37], Emotion Transformer (EmT) [38], and DBConformer [39]. Overall, LEPST-RBFNet achieved the best performance among the compared methods, with an average accuracy of 73.60 ± 9.90%.

Compared with the conventional machine learning methods, LEPST-RBFNet achieved a higher average classification accuracy under the same LOBO validation protocol. Common spatial pattern (CSP)-based methods [40,41,42] learn spatial filters to extract discriminative EEG variance patterns, while the discrete wavelet transform (DWT)-based method [43] uses wavelet feature extraction followed by a distance-based k-nearest neighbors (KNN) classifier. These methods rely on predefined spatial, spectral, or time-frequency feature representations, which may be insufficient to fully characterize the distributed EEG responses associated with familiar and unfamiliar face recognition. In contrast, LEPST-RBFNet introduces learnable scale-power parameters, allowing task-specific time-frequency feature transformations to be optimized jointly with the classifier.

Compared with the evaluated deep-learning-based methods, LEPST-RBFNet achieved the highest average accuracy. Among these deep learning models, EmT [38] obtained the best performance, with an average accuracy of 67.70 ± 10.87%. LEPST-RBFNet further improved this best deep learning result by 5.90%. Although deep-learning-based models can automatically learn discriminative representations, they usually contain more trainable parameters and may require larger datasets to achieve stable generalization. In the present EEG-based familiar and unfamiliar face classification task, the number of participants and trials was limited, which may restrict the advantage of large-capacity deep models. In contrast, LEPST-RBFNet uses a structured architecture based on local standard-deviation features, learnable entropy-power-inspired scaling, and prototype-based RBF classification, which may be more suitable for few-shot EEG feature learning.

It is also worth noting that FBDE + SVM [15] achieved a competitive average accuracy of 72.35 ± 10.67%, which was close to the performance of LEPST-RBFNet. However, unlike FBDE + SVM [15], which relies on fixed filter-bank differential entropy features and an external classifier, LEPST-RBFNet integrates adaptive feature transformation and prototype-based nonlinear classification into a unified trainable framework. Therefore, the proposed method provides not only competitive classification performance but also model-derived time-frequency scaling parameters for interpretation.

The subject-wise accuracies in Table 7 were further analyzed using paired Wilcoxon signed-rank tests. The results showed that LEPST-RBFNet achieved statistically significant improvements over most compared methods (p<0.05) and a marginally significant improvement over FBDE + SVM proposed by Liu et al. [15] (p=0.0703).

## 5. Conclusions

This study proposed LEPST-RBFNet for EEG-based familiar and unfamiliar face classification. The model combines time-frequency local standard-deviation features, a Learnable Entropy-Power Scaling Transform, and an RBF prototype classifier. The LEPST layer constructs an entropy-power-inspired learnable nonlinear transformation under the approximate Gaussian assumption of filtered EEG segments, while the RBF layer maps high-dimensional EEG features into a prototype-similarity space for classification. Experimental results demonstrated that LEPST-RBFNet achieved the highest average classification accuracy of 73.60%. Comprehensive ablation studies with extensive visualization and interpretability analysis verified the effectiveness and interpretability of the proposed method. These results suggest that entropy-power-inspired nonlinear transformation and prototype-based classification provide a promising framework for EEG-based familiar and unfamiliar face recognition. Nevertheless, this study still has several limitations. The dataset contained ten participants, which may limit the generalizability and robustness of the results and restrict the full demonstration of the adaptive advantage of LEPST. In addition, the present evaluation was mainly based on the within-subject LOBO protocol, and cross-subject generalization to unseen participants remains to be further investigated. Future work will focus on evaluating LEPST-RBFNet on larger EEG datasets, improving cross-subject generalization, and further enhancing model robustness through adaptive prototype selection and spatial EEG modeling.

## Figures and Tables

**Table 1:** Network architecture details of LEPST-RBFNet.

Layer	Output Shape	Core Operation
Input Features	(59,33,3)	Local standard deviation
LEPST Transform	(59,33,3)	$\sigma^{\lambda }$
Reshape	(59,99)	Time-frequency flattening
Flatten	(5841)	Vectorization
Layer Norm	(5841)	Scale equalization
RBF Layer	(140)	$\exp(-\beta ∥x-c_{i}{∥}^{2})$
Linear Output	(1)	$w^{T}\phi +b$

**Table 2:** Detailed block-level classification results of LEPST-RBFNet.

No.	Block 1 (%)	Block 2 (%)	Block 3 (%)	Block 4 (%)	Block 5 (%)	Max (%)	Mean ± Std (%)
1	55.00	67.50	77.50	65.00	67.50	77.50	66.50 ± 8.02
2	85.00	97.50	82.50	97.50	97.50	97.50	92.00 ± 7.58
3	65.00	57.50	70.00	65.00	55.00	70.00	62.50 ± 6.12
4	82.50	90.00	82.50	75.00	95.00	95.00	85.00 ± 7.71
5	75.00	80.00	77.50	87.50	75.00	87.50	79.00 ± 5.18
6	75.00	62.50	92.50	77.50	82.50	92.50	78.00 ± 10.95
7	52.50	72.50	62.50	65.00	67.50	72.50	64.00 ± 7.42
8	80.00	72.50	60.00	57.50	60.00	80.00	66.00 ± 9.78
9	80.00	60.00	82.50	80.00	75.00	82.50	75.50 ± 9.08
10	65.00	57.50	67.50	80.00	67.50	80.00	67.50 ± 8.10
Mean ± Std	71.50 ± 11.50	71.75 ± 13.80	75.50 ± 10.26	75.00 ± 12.13	74.25 ± 13.95	83.50 ± 9.37	73.60 ± 9.90

**Table 3:** Performance comparison among different classification heads using LEPST features (%).

Subject	Linear	ReLU	GELU	RBF
S1	66.00	67.50	64.00	66.50
S2	90.50	90.00	90.50	92.00
S3	60.50	62.50	60.50	62.50
S4	84.00	85.00	86.00	85.00
S5	81.00	80.50	79.00	79.00
S6	63.50	72.50	70.50	78.00
S7	57.00	62.00	63.50	64.00
S8	62.50	63.50	65.00	66.00
S9	68.50	74.00	73.50	75.50
S10	67.00	60.00	60.00	67.50
Avg.	70.05 ± 11.17	71.75 ± 10.52	71.25 ± 10.79	73.60 ± 9.90

**Table 4:** Performance comparison among different RBF prototype initialization strategies (%).

Subject	Random Samples	Global K-Means	Classwise K-Means	Farthest Point
S1	65.00	65.50	66.00	66.50
S2	92.00	92.00	92.00	92.00
S3	61.00	61.50	61.50	62.50
S4	85.00	85.50	84.50	85.00
S5	79.50	79.50	80.00	79.00
S6	78.50	77.00	76.50	78.00
S7	64.00	63.00	64.00	64.00
S8	66.00	66.50	66.00	66.00
S9	74.00	74.00	75.00	75.50
S10	66.50	66.00	66.00	67.50
Avg.	73.15 ± 10.32	73.05 ± 10.31	73.15 ± 10.10	73.60 ± 9.90

**Table 5:** Ablation study on learnable components of LEPST-RBFNet (%).

Subject	Fixed LEPST Parameters	Fixed RBF Centers	Proposed
S1	68.50	66.00	66.50
S2	92.00	91.50	92.00
S3	62.00	59.00	62.50
S4	83.50	76.50	85.00
S5	80.00	76.00	79.00
S6	76.50	72.00	78.00
S7	63.50	63.00	64.00
S8	65.50	64.00	66.00
S9	74.50	72.00	75.50
S10	66.50	61.00	67.50
Avg.	73.25 ± 9.80	70.10 ± 9.73	73.60 ± 9.90

**Table 6:** Performance comparison with different entropy measures under the LOBO validation protocol.

Entropy Measure	Accuracy (%)
Shannon Entropy	60.10 ± 9.88
Amplitude Entropy	55.50 ± 9.43
Incremental Entropy	52.83 ± 10.67
Differential Entropy	73.60 ± 9.90

**Table 7:** Comparison with representative EEG feature-extraction and classification methods evaluated on the familiar and unfamiliar face EEG dataset.

No.	Author	Method	Accuracy (%)
1	Novi et al. [40]	SBCSP	60.50 ± 10.90
2	Ang et al. [41]	FBCSP	62.12 ± 12.90
3	Liu et al. [42]	DivCSP + SVM	65.25 ± 10.05
4	Bardak and Temurtaş [43]	DWT + KNN	64.25 ± 7.78
5	Schirrmeister et al. [21]	ShallowConvNet	64.10 ± 12.70
6	Lawhern et al. [22]	EEGNet-8,2	62.00 ± 12.12
		EEGNet-4,2	58.50 ± 11.66
7	Liao et al. [36]	EEGEncoder	51.75 ± 2.69
8	Klein et al. [37]	PBT	51.25 ± 6.87
9	Ding et al. [38]	EmT	67.70 ± 10.87
10	Wang et al. [39]	DBConformer	60.55 ± 9.28
11	Altaheri et al. [44]	TCFormer	56.67 ± 13.66
12	Yang and Modesitt [45]	ViT2EEG	65.50 ± 8.72
13	Izzuddin et al. [46]	EEGNet + SincNet	60.50 ± 11.49
14	Riyad et al. [47]	MI-EEGNet	64.15 ± 11.71
15	Liu et al. [48]	Spatial-Temporal Self-Attention CNN	66.55 ± 12.13
16	Liu et al. [15]	FBDE + SVM	72.35 ± 10.67
17	This Work	LEPST-RBFNet	73.60 ± 9.90

## Data Availability

The raw data supporting the conclusions of this article will be made available by the authors on request.

## Abbreviations

The following abbreviations are used in this manuscript:

BCE	Binary Cross-Entropy
BCI	Brain-Computer Interface
CNN	Convolutional Neural Network
CSP	Common Spatial Pattern
DBConformer	Dual-Branch Convolutional Transformer
DivCSP	Diversity Common Spatial Pattern
DWT	Discrete Wavelet Transform
EEG	Electroencephalography
EEGEncoder	EEG Encoder
EmT	Emotion Transformer
ERP	Event-Related Potential
FBDE	Filter-Bank Differential Entropy
FBCSP	Filter-Bank Common Spatial Pattern
GELU	Gaussian Error Linear Unit
ICA	Independent Component Analysis
KNN	k-Nearest Neighbors
LEPST	Learnable Entropy-Power Scaling Transform
LN	Layer Normalization
LOBO	Leave-One-Block-Out
MI-EEGNet	Motor Imagery EEGNet
PBT	Patched Brain Transformer
RBF	Radial Basis Function
ReLU	Rectified Linear Unit
SBCSP	Sub-Band Common Spatial Pattern
SVM	Support Vector Machine
TCFormer	Temporal Convolutional Transformer
t-SNE	t-distributed Stochastic Neighbor Embedding
ViT2EEG	Vision Transformer for EEG
